# A response to Gadarowski’s letter to the editor

**DOI:** 10.1186/1477-7525-11-53

**Published:** 2013-04-11

**Authors:** Isabella Piva, Angela Graziano, Giuseppe Lo Monte, Stefano Caracciolo, Roberto Marci

**Affiliations:** 1 Department of Morphology, Surgery and Experimental Medicine, Section of Obstetrics and Gynecology, University of Ferrara, Ferrara, Italy; 2 Department of Experimental and Clinical Medicine, Section of General and Clinical Psychology, University of Ferrara, Ferrara, Italy

**Keywords:** Infertility, Psychology, Sexuality, Sexual dysfunction, Assisted reproductive technology, Sexual disorders, Sexual behavior, Psycho-sexology

## Abstract

This discussion is meant to examine the issues raised by Gadarowski in a recent Letter to the Editor.

This is a reply to http://www.hqlo.com/content/pdf/1477-7525-11-52.pdf.

## To the Editor

By emphasizing the strengths and limitations of our previous work Gadarowski highlighted the major importance of this research field and paved the way for interesting considerations.

Since sexual disturbances arising from fertility treatment threaten the success of assisted reproductive tecniques (ART) as well as the couple’s relationship, we aimed to detect such disorders in order to optimize clinical outcomes. Previous works mainly considered the long term consequences of involuntary childlessness: these can hardly be distinguished from the “natural” decline in sexual activity in long-term relationships [1] and are scarcely modifiable. Thus we chose the first steps of infertility diagnosis and treatment as a possible interventional area and we focused on the sexual dysfunctions and emotional stress that can occur in this short period of time. By detecting these problems at the very beginning of the clinical management it could be possible to solve them before they consolidate and become permanent. For this reason a longitudinal study goes beyond the objectives of our previous work, even though it represents an interesting suggestion for future research. However, a longitudinal study on personality and sexuality of infertile couples compared to fertile controls raises some issues. Firstly, since psycho-sexual counselling helps the couples to cope with emotional stress and sexual disorders [2], it would be unethical to detect such disturbances without providing the corresponding treatment. Secondly, compliance to such a long-term study is hardly achieved and the predictable high rate of drop-outs further extends the period of the research, that can result time-consuming. Of course the control group chosen should have included couples intentionally looking for pregnancy, but this point had explicitly been mentioned as a limitation of our study and will be improved in future works. As to the use of FSFI and IIEF, they represent well-established instruments to assess the main aspects of sexual functioning [3-5]. In particular, the subscale of sexual desire and pleasure could account for the recreational value of sexual intercourse. The term “Sex by the clock” does not mean a shorter duration of sexual intercourse, it indicates, instead, the habit of targeting sexual activity in the fertile days. Thus, the time spent on foreplay may not be affected in infertile couples. Additionally, the duration of foreplay does not necessarily account for eroticism in the couple, as it can depend on the pace of individual lifestyle and it is influenced by several factors, like the duration of the relationship [1], the desire to satisfy the partner’s wishes, the level of sexual communication between the partners, their cultural role and personal preferences [6]. Better, other aspects of the sexual interaction (e.g., expressions of affection, the nature of the sexual activities, the effectiveness of stimulation) are more predictive of sexual satisfaction than the duration of the sexual encounter [7]. Moreover, the typology of sexual intercourse could account for the distinction between procreative (complete coitus) and recreational sex (sexual-erotic activity that does not include complete coitus), as proposed by Quattrini et al. [8]. The evaluation of such aspects requires specially designed questionnaires investigating the intimate area, but they could be perceived a sintrusive, thereby limiting the response rate. Additionally, these instruments should be validated on an Italian sample and translated into Italian, otherwise they could cause biases. Infertility is a complex disease with sexual, emotional, social and marital repercussions. Even though the detection and management of such problems would be beneficial, this discussion highlights the difficulty in investigating and scientifically measuring them.

## Abbreviations

ART: Assisted reproductive technology; FSFI: Female sexual function index; IIEF: International index of erectile function.

## Competing interests

The authors declare that they have no competing interests.

## Authors’ contributions

All authors equally contributed to the drafting of the letter. All authors read and approved the final version of the manuscript.
